# Glioblastoma: A Multidisciplinary Approach to Its Pathophysiology, Treatment, and Innovative Therapeutic Strategies

**DOI:** 10.3390/biomedicines13081882

**Published:** 2025-08-02

**Authors:** Felipe Esparza-Salazar, Renata Murguiondo-Pérez, Gabriela Cano-Herrera, Maria F. Bautista-Gonzalez, Ericka C. Loza-López, Amairani Méndez-Vionet, Ximena A. Van-Tienhoven, Alejandro Chumaceiro-Natera, Emmanuel Simental-Aldaba, Antonio Ibarra

**Affiliations:** Centro de Investigación en Ciencias de La Salud (CICSA), Facultad de Ciencias de la Salud, Universidad Anáhuac México Campus Norte, Lomas Anáhuac, Huixquilucan 52786, Mexico; felipe.esparzas69@anahuac.mx (F.E.-S.); renatamurguiondo@gmail.com (R.M.-P.); gabriela.canohe@anahuac.mx (G.C.-H.); mariafrenandabg19@gmail.com (M.F.B.-G.); cristyloza01@gmail.com (E.C.L.-L.); 0238184@up.edu.mx (A.M.-V.); ximena.vantienhovenh@universidad.anahuac.mx (X.A.V.-T.); alejandro.chumaceiro@anahuac.mx (A.C.-N.); emmanuel.simental@anahuac.mx (E.S.-A.)

**Keywords:** glioblastoma, immunotherapy, stem cells, oncolytic virotherapy

## Abstract

Glioblastoma (GBM) is the most aggressive primary brain tumor, characterized by rapid progression, profound heterogeneity, and resistance to conventional therapies. This review provides an integrated overview of GBM’s pathophysiology, highlighting key mechanisms such as neuroinflammation, genetic alterations (e.g., EGFR, PDGFRA), the tumor microenvironment, microbiome interactions, and molecular dysregulations involving gangliosides and sphingolipids. Current diagnostic strategies, including imaging, histopathology, immunohistochemistry, and emerging liquid biopsy techniques, are explored for their role in improving early detection and monitoring. Treatment remains challenging, with standard therapies—surgery, radiotherapy, and temozolomide—offering limited survival benefits. Innovative therapies are increasingly being explored and implemented, including immune checkpoint inhibitors, CAR-T cell therapy, dendritic and peptide vaccines, and oncolytic virotherapy. Advances in nanotechnology and personalized medicine, such as individualized multimodal immunotherapy and NanoTherm therapy, are also discussed as strategies to overcome the blood–brain barrier and tumor heterogeneity. Additionally, stem cell-based approaches show promise in targeted drug delivery and immune modulation. Non-conventional strategies such as ketogenic diets and palliative care are also evaluated for their adjunctive potential. While novel therapies hold promise, GBM’s complexity demands continued interdisciplinary research to improve prognosis, treatment response, and patient quality of life. This review underscores the urgent need for personalized, multimodal strategies in combating this devastating malignancy.

## 1. Introduction

Glioblastoma (GBM), a WHO grade IV glioma, represents the most aggressive and prevalent primary brain tumor of astrocytic origin, with a 5-year survival rate of only 7.2% [1,2]. As the most common malignant brain tumor, GBM accounts for approximately 54% of all gliomas and 16% of all primary brain tumors [2]. Incidence rates vary worldwide, ranging from 0.59 to 3.69 per 100,000 persons, with the highest rates observed among malignant primary brain tumors [3]. Primarily affecting older adults, with a median diagnosis age of 64, it peaks in incidence among individuals aged 75 to 84 [2]. Rates are notably higher in men than in women, and incidence is elevated among Caucasians and non-Hispanics compared to other ethnic groups. It exhibits rapid progression, high infiltration into surrounding brain tissue, and frequent necrosis and vascular proliferation [4,5,6].

The tumor primarily arises in the supratentorial regions of the brain, with the frontal lobe being the most common location, followed by overlapping sites across multiple lobes, the temporal lobes, and the parietal lobe [7]. While extracranial metastasis is rare, the lungs and pleura are the most likely sites when it occurs [8,9]. The only confirmed risk factors for GBM are ionizing radiation and certain genetic syndromes, with no established link to routine diagnostic radiation exposure [10,11].

The standard approach to GBM treatment typically begins with a biopsy, tumor debulking, or maximal resection based on the tumor’s location and the patient’s clinical condition. The extent of resection is a crucial prognostic factor, with a more comprehensive removal of tumor tissue correlating with better outcomes. For patients who are fit for surgery, the aim is to achieve maximum cytoreduction while minimizing neurological risks. Following surgical intervention, standard therapy involves chemoradiotherapy, usually with temozolomide (TMZ); however, despite these aggressive measures, the 5-year survival rate remains 5–10% in the United States [1,2]. Moreover, recurrence is almost inevitable due to challenges such as incomplete resection, GBM’s genetic heterogeneity, the protective blood–brain barrier (BBB), and the tumor’s immunosuppressive microenvironment. This complex environment not only limits therapeutic efficacy but also poses significant challenges for durable treatment responses [12].

To address these limitations, recent advances have focused on innovative therapies beyond traditional approaches. Immunotherapy, including the use of checkpoint inhibitors, cancer vaccines, and chimeric antigen receptor (CAR) T cell therapies, is now a promising area of development, with the potential to target GBM’s unique immune landscape. Additionally, oncolytic virotherapy (OV) offers a novel strategy to selectively infect and destroy tumor cells while stimulating an antitumor immune response [13]. Personalized medicine, exemplified by individualized multimodal immunotherapy (IMI), tailors treatments to each patient’s tumor immune profile, utilizing immunogenic cell death (ICD) mechanisms to enhance the body’s natural defenses. Advances in nanotechnology-based therapies, such as NanoTherm therapy and precision radiotherapy techniques, further complement these immunotherapies, aiming to overcome barriers posed by GBM’s resistant and heterogeneous structure [14].

Despite advances in multimodal treatments, GBM remains one of the most challenging cancers to treat, with a median survival rate of only 14.6 months [15]. This dismal prognosis underscores the need for continuous research in pharmacological and non-pharmacological treatment strategies to improve outcomes for GBM patients.

## 2. GBM Pathophysiology

The pathophysiology of GMB is complex, as it involves various processes.

### 2.1. Neuroinflammation

The pathophysiology of GBM includes multiple cellular and genetic alterations that play an essential role in the progression and aggressiveness of the disease and can be associated with a poor prognosis [16]. Neuroinflammation is caused in part by the involvement of growth factors, such as IL-6, IL-1β, and TNF-α, that activate microglia to attract macrophages to the tumor, releasing chemokines and cytokines that generate a proinflammatory feedback loop [17].

The central nervous system (CNS) was previously considered immunologically privileged due to the presence of the BBB, which is a protective layer composed of endothelial cells with tight junctions that prevents the entry of pathogens and toxins; previously, it was thought that it did not allow the passage of any antigen-presenting cell (APC) and did not have a lymphatic drainage system [18]. Currently, studies carried out in mouse and human models demonstrate that the CNS has a functional lymphatic system within the meninges that drains to the cervical lymph nodes and resident APC cells [19,20].

Moreover, it has been observed that the BBB suffers modifications when an adjacent disease, such as cancer, is present. Inflammation related to GBM causes the loss of BBB impermeability, allowing a greater passage of proinflammatory cells into the brain. This leads to chronic inflammation and a neuroinflammatory response that directly promotes tumorigenesis, leading to both tumor growth and the generation of a survival tumor microenvironment (TME) by the surrounding stroma [20,21]. Similarly, GBM is characterized by causing tissue necrosis influenced by its inflammatory TME [17].

Although neuroinflammation is a common manifestation in GBM, its existence in the areas contralateral to the primary tumor does not have an understood pathophysiology [20,22]. A study conducted by Bartos et al. analyzed the expression of neuroinflammation caused by the primary tumor in distant brain regions through a non-invasive PET study with translocator protein (TSPO-PET). They concluded that contralateral neuroinflammation functions as a biomarker for a more precise prognosis for GBM [23].

### 2.2. Genetics

Although the genetic etiology of GBM is not specific, it has been demonstrated that this tumor can arise from various alterations in different genes that regulate essential cellular processes. This is done through common signaling pathways that induce or inhibit cell proliferation, apoptosis, and differentiation, such as Rb, the P53 pathway, and the receptor tyrosine kinase/Ras/PI3K pathway [24].

The genes that are manifested in the pathophysiology of GBM include EGFR, Cyclin-Dependent Kinase 4 (CDK4), MDM4 Regulator of p53 (MDM4), and platelet-derived growth factor receptor alpha (PDGFRA). The EGFR gene has the ability to generate certain intragenic mutations, such as EGFRvIII, that create oncogenic proteins, which remain in an active state and secondarily cause the activation of the PI3K-AKT pathway [25]. The amplified and mutated PDGFRA gene contributes to uncontrolled signaling that promotes tumor growth [26]. CDK4 and MDM4 promote cell cycle progression and evasion of apoptosis, exacerbating tumor aggressiveness [27]. In pathophysiological terms, the interaction between these genetic alterations reinforces the intratumoral heterogeneity and resistance to treatment that is characteristic of GBM. Intragenic mutations in EGFR and PDGFRA produce functionally active variants that not only enhance cell division, but also modify the TME, promoting tumor invasion and facilitating angiogenesis [25,26,27,28].

Another gene that has a great influence on the inflammatory TME of GBM is the Triggering Receptor Expressed on Myeloid Cells 1 (TREM1). This gene is mainly involved in perinecrotic and hypoxic areas of the tumor and is present in myeloid cells such as macrophages and neutrophils; it is also activated by damage-associated molecules (DAMS) released by tumor cells [29]. TREM1 signaling promotes the production of inflammatory cytokines and fusogenic proteins, favoring tumor progression, heterogeneity, and resistance to treatment. Different studies have indicated that inhibitors such as SRI42127 can reduce the activation of TREM1 and limit the migration of myeloid cells to the site of the tumor, suggesting a promising strategy to control the progression of GBM [30].

The mutation of the SWI/SNF-related, matrix-associated, actin-dependent regulator of chromatin, subfamily b, member 1 gene (SMARCB1), which is located in chromosome 22q11.1, has been linked to the development and early appearance of GBM. This process occurs through the interaction with other genes such as PDGFRA and HRAS, responsible for encoding an essential subunit of the SWI/SNF chromatin remodeling complex, known for its tumor suppressor function through epigenetic regulation of gene transcription [31]. In a study of familial GBM, it was observed that heterozygous and homozygous SMARCB1 mutations were associated with a diagnosis at a significantly younger age, suggesting that these genetic alterations accelerate tumor progression, promoting cell proliferation through pathways such as PI3K and MAPK [32].

Although these alterations have the potential to function as biomarkers of GBM, their clinical impact and association with prognosis remain the subject of research, underscoring the need to integrate this information for the development of personalized medicine.

### 2.3. Microbiome

The influence of the gut microbiota has gained importance in the last decade, with increasing evidence of its role in the pathophysiology of various metabolic diseases, such as brain tumors [33]. The intestine communicates with the brain through a bidirectional pathway known as the gut–brain axis, which is essential for maintaining homeostasis through the absorption of nutrients, synthesis of vitamins, hormonal functions, and carbohydrate fermentation, among others. New evidence suggests that the microbiome may influence brain tumor metabolism by promoting or inhibiting the malignant progression of GBM, consequently altering the epigenome of glioma cells through deregulation of its metabolites [34,35]. Despite limited evidence, the potential association has been demonstrated.

Moreover, in a study carried out by Zeng et al., a Mendelian randomization analysis demonstrated that the bacteria Prevotella 7, Ruminococcaceae, Faecalibacterium, and Anaerostipes may have implications in GBM screening and treatment [36]. Similarly, Patrizz et al. demonstrated that glioma implantation in mice is sufficient to induce gut microbiota dysbiosis, supporting the hypothesis about an interaction between the tumor and the microbiota [37]. Furthermore, in a study carried out in 2021 by Wen et al., a greater population of Fusobacteria, Proteobacteria, and Bacteroidetes was observed in patients with malignant tumors when compared to patients with benign tumors, as well as lower concentrations of Firmicutes and Actinobacteria in patients with brain tumors [34,38]. Bifidobacterium and Lactobacillus, through fermentation, produce short-chain fatty acids such as butyrate, propionate, and acetate. They found that these molecules cross the BBB without major problems, and concluded that they may be able to modulate the function of microglia and neurogenesis [34]. For example, butyrate can inhibit tumor growth and induce cell apoptosis through molecular mechanisms, demonstrating efficacy against colon, prostate, and liver cancer [39,40,41]. Therefore, it is now thought that an alteration in short-chain fatty acids can lead to the accumulation of toxic components in the brain that could contribute to the development of tumors [34].

The mentioned findings suggest that dysbiosis, characterized by increased Fusobacterium and decreased Bifidobacterium, in glioma patients, could serve as an early indicator to differentiate between benign and malignant tumors, as well as a key distinguisher between healthy individuals and cancer patients. This distinction could further be supported by certain microbial patterns, such as an overexpression of potentially pathogenic bacteria associated with malignancy (for example, Shigella and Escherichia) and a reduction of bacteria commonly found in healthy individuals (for example, Lachnospira) [34,42,43,44].

Despite the effort to find a direct relationship, there are still a limited number of studies on the role of microbiota in tumor diseases. What is certain is that intestinal dysbiosis generates chronic inflammation, which is associated with tumorigenesis, including GBM [34].

### 2.4. Microenvironment

The GBM TME is a component that greatly influences tumor progression and the development of effective therapies. It is made up of immune cells such as microglia, tumor-associated macrophages, T and B lymphocytes, and natural killer (NK) and dendritic cells. It creates an immunosuppressive environment that facilitates evasion of the immune system by the tumor and plays a role in treatment resistance [45,46,47]. For example, microglia and macrophages can switch from a proinflammatory (M1) to a protumor (M2) phenotype, favoring tumor growth [48].

Additionally, the TME includes non-immune cells, such as astrocytes, pericytes, endothelial cells, and neurons, which interact with tumor cells to promote their growth and dissemination [45,49]. Furthermore, the TME of GBM is distinguished by an extracellular matrix characterized by an overexpression of collagen and fibrillar networks that facilitate tumor invasion and immune exclusion, hindering the effectiveness of immunotherapies [45,49].

All of these components are important when creating a hypoxic environment in GBM, a process that occurs naturally due to rapid tumor proliferation that outpaces oxygen supply, leading to diffuse hypoxia. Additionally, it can be exacerbated by microscopic intravascular thrombosis, which limits blood flow and further contributes to perfusion hypoxia [50]. This hypoxic factor promotes angiogenesis, tumor invasion, and metabolic reprogramming, which facilitates the survival and proliferation of tumor cells [46,51,52].

Hypoxia also influences tumor plasticity and heterogeneity, promoting a stem cell-like state in glioma cells, which is associated with resistance to standard therapies [53]. Regarding immune cells, hypoxia creates an immunosuppressive environment that inhibits the activity of cytotoxic T cells and favors the proliferation of immunosuppressive cells, such as tumor-associated macrophages in their M2 phenotype [54,55].

Advances in understanding the TME of GBM have opened new perspectives for the development of more effective treatments. Immune cells, such as tumor-associated macrophages and microglial cells, have an essential impact in promoting tumor progression and immune evasion. Therefore, these cells are promising therapeutic targets; consequently, strategies to make the TME more permissive to immunotherapies are currently under development [56,57].

### 2.5. The Immunologic Role of GBM

GBM is challenged by its immunosuppressive tumor microenvironment, where glioma-associated microglia (GAMs) play a key role in modulating the immune response. These cells can transform into M1 or M2 phenotypes, depending on the cytokines and chemokines present, with M1 inhibiting the tumor and M2 promoting its growth [58]. GAMs in GBM exhibit great plasticity, favoring immunosuppressive functions, such as the production of anti-inflammatory cytokines and the expression of PD-L1. This protein aids neoplastic cells in evading the immune system, which promotes tumor progression, angiogenesis, and activation of immune checkpoints [58,59].

Neoplastic cells, including glioma stem cells, are a key factor in the suppression of the immune system through various mechanisms, such as secreting immunosuppressive cytokines, attracting regulatory T cells (Tregs), and the expression of immune checkpoints [60]. On the other hand, macrophages are considered promoters of tumor growth due to their proangiogenic and immunosuppressive effects. Among these, myeloid-derived suppressor cells (MDSCs) significantly contribute in modulating the immune response [61].

### 2.6. Molecular Mechanisms Implicated in GBM

The molecular mechanisms involved in GBM have been the subject of extensive studies, highlighting three main processes: (1) DNA methylation consists of the addition of methyl groups to specific DNA bases, which can lead to gene silencing. In the context of GBM, abnormal methylation of tumor suppressor genes facilitates the proliferation of malignant cells. (2) Histone modification that alters the accessibility of DNA to transcription factors results in the activation of oncogenes and deregulation of key genes. (3) Chromatin remodeling that modifies the structure of the complex formed by DNA and histones, altering its accessibility and contributing to gene deregulation [61].

### 2.7. The Role of Gangliosides in GBM

Gangliosides play a multifaceted role in the pathophysiology of GBM, with the disialoganglioside GD3 emerging as the most prominent. GD3 is highly expressed in GBM stem-like cells, where it is essential for maintaining stemness, self-renewal, tumorigenicity, and chemoresistance [62,63]. It also contributes to tumor aggressiveness by promoting proliferation, invasion, and motility. These effects are mediated through the activation of key oncogenic signaling pathways, including Akt, Erk1/2, and focal adhesion kinase, as well as through the formation of signaling complexes within lipid rafts involving platelet-derived growth factor receptor α (PDGFRα) and Yes kinase, which further amplify malignant signaling [64,65,66]. Inhibition of GD3 or its synthesizing enzyme, GD3 synthase (ST8SIA1), impairs these tumor-promoting processes, reduces tumor growth, and enhances sensitivity to temozolomide, highlighting GD3 as a compelling therapeutic target [62,63]. Moreover, GBM exhibits a distinct ganglioside profile characterized by elevated levels of simple gangliosides, particularly GD3 and its O-acetylated forms, which distinguish tumor tissue from normal brain and may serve as useful molecular markers or therapeutic entry points [67,68,69].

### 2.8. The Role of Ceramides and Sphingolipids in GBM

Ceramides and sphingolipids are crucial players in GBM pathogenesis due to their roles in the sphingolipid rheostat, a dynamic system that regulates the balance between pro-apoptotic ceramide and pro-survival sphingosine-1-phosphate (S1P) [70,71]. In GBM, this balance is disrupted, with elevated S1P and reduced ceramide levels, leading to enhanced tumor cell survival, proliferation, invasiveness, angiogenesis, and evasion of apoptosis [72,73].

## 3. Diagnostic Methods of GBM

The diagnosis of GBM involves clinical evaluation, imaging, and histopathology. In recent years, innovative techniques such as immunohistochemistry, biomarkers, and liquid biopsy have emerged as promising diagnostic tools.

### 3.1. Clinical Diagnosis

The initial diagnostic suspicion of GBM arises from its clinical presentation [74]. New-onset headaches in patients older than 50 years, particularly with atypical features such as progressive severity, changes in presentation, or unilateral localization, are warning signs for the suspicion of an intracranial tumor [75]. Up to 71% of patients diagnosed with GBM report headaches [75,76]. Other neurological manifestations include increased intracranial pressure, seizures, nausea, vomiting, fatigue, personality changes, papilledema, and vision disturbances [77]. The clinical presentation of GBM can appear months or even years after tumor onset, with symptoms varying based on its size, location, and characteristics [76,77].

### 3.2. Imaging

When GBM is clinically suspected, imaging studies are essential for confirmation. Magnetic resonance imaging (MRI) is the modality of choice due to its high sensitivity in identifying tumor characteristics [74,77]. The prevalence of brain tumors found incidentally during neuroimaging is 0.7%, with only 0.05% being GBM [78]. MRI typically shows three distinct regions: a non-enhancing central necrotic core on T1-weighted images, an active tumor area with contrast enhancement, and a peripheral zone with hyperintensities on T2/FLAIR sequences due to edema or peritumoral invasion [79]. Invasion into deep white matter and the corpus callosum is common, often presenting as multifocal lesions with small areas of enhancement and regional necrosis [80].

### 3.3. Histopathology

The definitive diagnosis of GBM relies on histopathological examination of the tumor following surgical resection. Tumors are typically around 4 cm in size, with key features including necrosis and microvascular proliferation, along with anaplasia, cellular invasion, pleomorphism, increased mitotic activity, and high cellular proliferation. While not pathognomonic, these findings indicate tumor aggressiveness [81,82]. Necrosis and microvascular proliferation are hallmark features of GBM and fundamental to its diagnosis [83].

### 3.4. Immunohistochemistry and Biomarkers

Immunohistochemical (IHC) analysis is crucial for diagnosing GBM and is a key component of the 2016 WHO classification [84]. Glial fibrillary acidic protein (GFAP), a specific marker for astrocytes, confirms the glial origin of GBM. Loss of GFAP expression may indicate a more undifferentiated and aggressive tumor [74]. Additionally, GBM frequently expresses vimentin and S100, further supporting its glial nature [85].

Mutations in the isocitrate dehydrogenase (IDH) genes, particularly IDH1 R132H, are central to GBM classification. IDH mutations, common in secondary GBM, result in the production of 2-hydroxyglutarate, a metabolite implicated in tumorigenesis [81,84]. These mutations are absent in most primary GBMs (IDH-wild), which represent the majority of cases. Rapid IHC staining techniques enable efficient identification of IDH1 mutations, aiding diagnosis [86].

IDH mutations are common in secondary GBMs and rare in primary GBMs, which are predominantly IDH-wild [84]. The presence of IDH-wild status, alongside classical molecular features, is sufficient to confirm GBM diagnosis, even in cases with atypical histopathological findings [83,86].

The methylation status of the MGMT (O6-methylguanine-DNA methyltransferase) promoter is a critical prognostic factor. Hypermethylation silences MGMT, reducing DNA repair activity and increasing tumor sensitivity to temozolomide (TMZ) chemotherapy, leading to better outcomes [87]. Conversely, unmethylated MGMT promotes resistance to alkylating agents, correlating with poor prognosis [88,89].

The proliferation marker Ki67 is another key biomarker, reflecting tumor growth activity. Mitotic rates in GBM range from 15 to 20% and can exceed 50% in aggressive cases, with higher Ki67 levels often observed in IDH-wild GBMs. Elevated Ki67 has been associated with improved survival outcomes [84,90].

ATRX gene mutations, which are more common in low-grade gliomas, are rare in GBM. Loss of ATRX expression in IHC tests aids in distinguishing GBM from other low-grade astrocytomas [84,90]. ATRX mutations often co-occur with IDH mutations in younger patients [91].

### 3.5. Liquid Biopsy

Traditionally, GBM diagnosis relies on histopathological evaluation after surgical resection or fine needle aspiration biopsy for unresectable tumors [74]. Recent advances in molecular diagnostics have introduced liquid biopsy as a complementary tool, enabling precision medicine approaches to GBM identification and monitoring [92]. Liquid biopsies analyze circulating biomarkers—such as circulating tumor DNA (cfDNA), messenger RNA (mRNA), and exosomes—in biological fluids like blood, cerebrospinal fluid (CSF), or urine [93].

The detection of cfDNA and tumor mRNA in CSF provides high sensitivity for identifying GBM-specific mutations and genetic alterations. This minimally invasive method offers an advantage over surgical resection or needle biopsy and can identify key mutations, such as IDH gene mutations or MGMT promoter methylation [94]. Liquid biopsy also allows for monitoring tumor burden, tracking treatment response, and detecting early signs of recurrence or resistance [95,96].

Exosome analysis is emerging as another diagnostic avenue. Tumor-derived exosomes, which contain proteins, lipids, and genetic material, offer insights into tumor heterogeneity and active signaling pathways in GBM [94]. These biomarkers provide real-time tumor profiling, potentially enhancing diagnostic accuracy and serving as an alternative to conventional biopsy [97].

While liquid biopsy is not yet a full substitute for traditional diagnostic techniques such as tumor biopsy or imaging, its ongoing development underscores its potential [98]. Its ability to provide a non-invasive diagnosis, track disease progression, and assess therapeutic efficacy makes it a promising tool, especially for cases involving unresectable, hard-to-reach, or recurrent GBM [99].

## 4. Treatment

When the diagnosis of GBM is made, multidisciplinary management is required. The current standard consists of maximal surgical resection, accompanied by concurrent radiation with temozolomide (TMZ), adjuvant chemotherapy with TMZ, and an oral alkylating chemotherapy agent. The goal of treatment is to improve symptoms, reduce medication consumption, and increase survival [4,100].

### 4.1. Conventional Treatment

#### 4.1.1. Surgical Approach

Surgical resection of GBM is complicated, given that these tumors are found in eloquent areas of the brain and are frequently invasive. Additionally, given the tumor’s nature, surgical excision may lead to a high recurrence rate and progression of the disease. However, there are prognostic factors in tumor resection such as extension, tumor size, and age [4]. The standard of treatment follows the steps of the Stupp Protocol, which consists of complete surgical resection of the tumor and subsequent use of radiotherapy (RT) for patients with a recent diagnosis of GBM [100,101]. However, in the elderly population, the biological age is taken into account for the approach selection, and it has been shown that maximal resection is the optimal alternative when the general health and functional status of the patients is adequate [102]. It is important to mention that the majority of GBM cases relapse near or within the initial location of the disease. For patients with recurrent GBM, there is currently no standard of care as treatment options including radiation, systemic therapy, and surgery. The most commonly used treatment alternative is the surgical approach, with a mean survival reported in the literature ranging between 6 and 17 months. The technique with the best prognosis is subtotal or total resection of the malignant lesion [103]. Wolbers et al. also mention that in patients with a recent diagnosis of GBM, an extensive and safe resection has a better prognosis and greater overall survival, suggesting that complete surgical resection of the tumor in early stages confers the best treatment option. Moreover, the use of fluorescence-guided agents such as 5-amino-levulinic acid (5-ALA) facilitates tumor detection during surgical procedures compared to conventional white-light surgery, as the molecule protoporphyrin IX (PpIX) accumulates in damaged tissue and emits fluorescence, thereby enhancing the rate of complete resection of GBM in approximately 65% of cases [4,104].

#### 4.1.2. Pharmacological Approach

Regarding the medical approach, there are multiple pharmacological alternatives; however, their purpose is to prevent progression; they do not confer a curative result. Antiangiogenic drugs began to be studied at the beginning of the 21st century, with the analysis of the role of vascular endothelial growth factor (VEGF) in the pathophysiology of GBM. However, after multiple studies and trials using VEGF, they demonstrated that its use, both in monotherapy and in combination, did not represent a benefit in the progression and survival of the disease, with bevacizumab being the most used drug [105,106,107].

##### TMZ and Alkylating Agents

TMZ is an oral alkylating agent that has adequate penetration into the CNS. It was approved in 1999 to treat malignant gliomas. It is a derivative of carbamazepine, and promotes methylation of the 06 position of guanine (N7-guanine and N3-adenine). The initial dose is usually 75 mg/m^2^ per day and is accompanied by 6 weeks of regional radiotherapy. The use of concurrent TMZ and RT increases the median overall survival from 14.6 months to a 2-year survival rate [107]. A study by Schaff et al. reports that the use of TMZ with radiotherapy in patients with glioblastoma significantly improves survival compared to patients treated with radiotherapy alone. Survival in patients treated with TMZ and radiotherapy at 2 years was 27.2%, and in patients treated only with radiotherapy, it was 10.9% at 5 years [77]. Meanwhile, Herrlinger et al. suggest that the use of TMZ with chemotherapy improves the survival of patients diagnosed with glioblastoma [108].

##### Paclitaxel

Another drug used for the treatment of GBM is paclitaxel (PTX), which is a chemotherapeutic that selectively attacks proliferating cells. A study by Wang et al., 2023. demonstrates that the use of PTX stimulates a macrophage-mediated immune response for the local approach to recurrent GBM. PTX filaments sensitize the tumor through a CD47-mediated blockade that promotes macrophage-mediated phagocytosis of tumor cells [109]. Another study carried out by Qu et al. 2023 concluded that albumin-bound paclitaxel has a synergistic inhibitory effect on GBM cells when combined with TMZ, so this combination suppresses GBM progression, increasing the survival of the study mice [110].

##### Exportin 1

Exportin 1 (XPO1) is a protein that has the function of transporting some messenger RNAs and approximately 220 proteins out of the nucleus to act as tumor suppressors, such as Rb1, p27, and P53, which are important markers for cancer prevention. Selective inhibitors of nuclear export (SINE) have demonstrated great efficacy in preclinical studies in animal models of GBM to promote apoptosis. Thus, Selinexor is an XPO1 inhibitor that has been advancing in clinical studies for patients with recurrent GBM [111]. A phase II study evaluating the safety and efficacy of oral Selinexor in recurrent GBM by Lassman et al. (2022) demonstrated that this drug is safe for the treatment of recurrent GBM [112].

##### Carmustine Wafers

Carmustine wafers are biodegradable polymers; few studies have shown prolonged survival in GBM patients with this alternative. However, one study showed that using carmustine increased overall survival to 8.7 months when compared to a group without wafers, with a 5.5-month survival. Regardless, more studies are needed to approve this treatment method [113]. On the other hand, a systematic review carried out by Zhang et al. (2014) reviewed the use of carmustine wafers in the treatment of GBM, where it was concluded that patients who received treatment with these wafers had improved survival, with no adverse effects [114].

##### Curcumin

Curcumin (diferuloylmethane) is a yellow pigment that comes from the rhizome of turmeric, which has an anti-inflammatory effect. In addition, it has some effect on growth factors that are associated with antitumor effects. Curcumin has been shown to have effects on malignant GBM cells by decreasing their number, promoting apoptosis, and enhancing the effects of radiation and chemotherapy. To date, there is extensive scientific evidence from high-quality in vitro data suggesting that the use of curcumin has the potential to complement RT and CT [115,116].

##### Ceramide Therapeutics

While ceramide promotes tumor suppression by triggering apoptosis and blocking proliferation, S1P acts as a tumor promoter, facilitating survival, migration, and vascular development. A shift toward S1P dominance is associated with more aggressive and higher-grade GBMs [72]. This imbalance is regulated by key metabolic enzymes—SPHK1, which catalyzes S1P production, and SGPP2, which degrades S1P [72].

Therapeutically, restoring the balance in favor of ceramide offers potential benefits. Inhibiting SPHK1 decreases S1P levels and has been shown to reduce angiogenesis in GBM models [72]. Enhancing ceramide accumulation makes GBM cells more responsive to TMZ, whereas resistance to TMZ has been linked to disrupted ceramide pathways, especially in tumors overexpressing EGFRvIII [117]. S1P further contributes to tumor resistance by regulating ceramide metabolism through the PI3K/Akt signaling pathway, which is frequently overactive in GBM, supporting both tumor progression and resistance to ceramide-driven apoptosis. Ceramides and lipid metabolism influence glioblastoma resistance, as ceramide is involved in temozolomide resistance in EGFR cells, and sphingosine-1-phosphate helps ceramide transport, promoting cell therapy; thus, these lipids represent promising therapeutic targets [118,119].

#### 4.1.3. Radiotherapy

Radiotherapy (RT) has been established as a standard therapeutic strategy for GBM since 2005, following a phase III clinical trial that validated its combination with adjuvant chemotherapy in the postoperative period. The treatment involves the administration of doses of 2 Grays (Gy) for a period of 6 weeks, with a target dose of 60 Gy. This modality has proven to be effective, especially in fractionated doses, which allows its use in patients over 65 years of age [120].

However, the use of RT is limited in recurrent gliomas, with greater efficacy as a palliative treatment in small recurrent tumors. The application of this treatment should take into account factors such as the patient’s previous history of radiation therapy, the location of the tumor, and the maximum allowable dose for the involved structures. Furthermore, advanced age (over 70 years of age) and a deteriorated functional status, as measured by the functional score scale for intensive care, are criteria that contraindicate the use of chemoradiotherapy in certain patients [120,121,122].

### 4.2. Non-Conventional Treatments

#### 4.2.1. Palliative Care

The incorporation of palliative care as part of the management of patients with glioblastoma has been widely discussed, with discrepancies found about the true impact it has on the patient depending on their age, the disease stage, the prognosis, and their personal desires. During GBM progression, the patient’s neurological integrity is severely affected, consequently influencing their decision-making ability regarding their clinical conditions [123]. Likewise, it is important to highlight that patients with GBM present a wide variety of neurological symptoms, including seizures, headaches, delirium, decreased consciousness, and aphasia in some cases, in addition to total dependence on third persons due to the motor comorbidities present in more than half of the patients [124]. Likewise, it has been studied that exposure to treatments such as radiotherapy and chemotherapy also promotes cognitive deterioration [125]. Thus, the combination of malignant characteristics specific to the tumors, the actual limited and invasive therapeutic options, and poor prognosis may contribute to the lower use of end-of-life care [126]. For these reasons, it is important to determine at what clinical stage the integration of palliative care would be most beneficial for their quality of life and their caregivers.

Unlike other types of cancer, physical activity in GBM is significantly decreased due to the disease’s proper neurological symptoms, as previously mentioned (fatigue and headache, mainly), making exercise an activity that is not only complex but also perceived as non-attractive to most patients [127]. Despite this, it has been documented in a variety of studies that physical activity has several specific benefits in GBM, such as improvement in the overall severity of symptoms, quality of life, improved patient-perceived mood, and strengthening of neurocognitive domains. However, in these studies, the most notable limitation is that it has only been applicable in patients with early diagnosis and with a slight clinical decline [128,129,130]. Given the above, the implementation of physical activity in most GBM patients presents complex results, given their neurological deterioration; in addition, currently, there is a lack of sufficient evidence to establish the specific benefits that can be provided to the clinical course of the disease.

#### 4.2.2. Diet Implementation

On the other hand, changes in the diet of patients with GBM have also been widely discussed in recent years. It has been well known that glucose is the brain’s, and neoplastic cells’, main source of energy. In addition, it has been documented that it increases proliferation, and growth factors similar to insulin play a role as carcinogenic promoters [131].

Following the implementation of the ketogenic diet—commonly acknowledged as a therapeutic strategy in GBM—glucose supply is decreased, which consequently promotes ketone bodies (acetoacetate and beta-hydroxybutyrate) to reach the brain as an alternative energy supply. These ketone bodies cross the BBB through monocarboxylate transporters and, with lactate, adjust to the energy metabolism of the brain. Despite the fact that non-neoplastic cells are capable of adjusting to this source of energy, GBM tumor cells lack mitochondria, and become incompetent to satisfy their energy needs; thus, their growth and proliferation are directly affected [132]. Likewise, it has been found in a previous study with animal models with GBM that this diet provides a synergistic effect combined with radiotherapy, demonstrating that it decreases the angiogenesis of tumor lesions [133]. The incorporation of the ketogenic diet as an anticancer adjuvant has been widely proposed. Despite this being mentioned, contradictory evidence has been shown about the real benefits that the present diet provides, and it has even been proposed that it may be counterproductive or may not generate a direct change in the prognosis or clinical course of GBM [134,135,136].

### 4.3. Future Perspectives

#### 4.3.1. Optune

In 2015, the United States Food and Drug Administration (FDA) approved Optune, a device that releases Tumor Treatment Fields (TTFs) and interrupts cell division, causing tumor cellular death [4]. TTFs are a novel non-invasive locoregional antineoplastic therapeutic option that utilizes low-intensity, intermediate-frequency, and alternating electric fields. This device utilizes electrodes that are placed on the patient’s shaved scalp. TTFs cause disruption of mitosis, resulting in plasma membrane contractions and instability, as well as the formation of plasma membrane blebbing. During metaphase, TTFs affect motility and assembly of intracellular macromolecules required for spindle formation, leading to chromosomal breakage and cell death. During anaphase, telophase, and cytokinesis, TTFs disrupt the polarity and alignment of several cellular structures, leading to an incomplete cytoplasmic separation and apoptosis [137].

Another mechanism of this device includes interference with septin fibers of proliferating tumor cells, in addition to causing endoplasmic reticulum stress, leading to autophagy. While this mechanism appears to be promising as a potential novel therapeutic alternative for GBM, the effects that this device may have on other intracellular proteins are still unknown; therefore, more research should be conducted in both cancer and normal cell lines [138]. A clinical trial was carried out in which Song et al. used TTFields in combination with temozolomide and radiotherapy for treating glioblastoma, which are low-frequency and -intensity electric fields. It was reported that it was not only a high-maintenance-rate, feasible, and well-tolerated treatment, but also, it did not cause severe toxicities [139].

#### 4.3.2. Immunological Therapy

In recent years, substantial progress has been made in the development of targeted immune therapies for the treatment of GBM. Immune checkpoint inhibitors, such as PD-1/PD-L1 antagonists, have been shown to be effective in restoring T cell function, allowing reactivation of the immune system to recognize and attack tumor cells. In addition, cell-based therapies, such as chimeric antigen receptor T cells (CAR-T), are being evaluated in preclinical and clinical trials, with encouraging results in GBM models [56]. A study by Bagley et al. demonstrates that bivalent CAR-T cells that are directed against EGFR and IL13Ra2 in recurrent glioblastoma is a safe therapy in humans, although it causes reversible neurotoxicity, so further studies are needed [140]. A case study reported by Brown et al. demonstrated that intrathecal administration of CAR-T cells against IL-13Ra2 induced a transient and complete response in a patient with recurrent multifocal glioblastoma, improving the patient’s quality of life by activating the endogenous immune system [141].

In addition, approaches that combine immune therapies with agents that modulate SMT are being explored. For example, the use of bioactive nanoparticles to block the signaling of the chemokine CXCL12/CXCR4, which plays a crucial role in tumor-associated myeloid cell mediated immunosuppression (TAMCs), has been shown to be effective [142]; inhibition of this pathway with nanoparticles co-administering a CXCR4 antagonist and an immune checkpoint inhibitor has been shown to reprogram the TME, reducing TAMCs and regulatory T cells, and increasing the proportion of activated CD8+ T cells. This approach not only enhances BBB penetration but also potentiates the antitumor immune response [143]. Similarly, the combination of nanoparticles with radiotherapy has been shown to induce immunogenic cell death and enhance the efficacy of immune checkpoint inhibitors, modulating SMT to favor systemic antitumor immunity [144].

The field of oncology has recently witnessed the emergence of novel therapeutic vaccines that are being evaluated for their potential in inducing a specific immune response against tumor antigens. A notable example is the DCVax-L vaccine, which utilizes the patient’s own dendritic cells to stimulate an immune response directed against the tumor. Preliminary studies have demonstrated encouraging results, suggesting the promise of this vaccine. Furthermore, the combination of immunotherapy with conventional therapeutic modalities, such as RT and QT, is under investigation to enhance the immune response and overcome the immunosuppression present in the tumor microenvironment. These advancements offer novel prospects for the treatment of GBM, though further research is necessary to optimize their efficacy and surmount the challenges inherent to the complexity of this tumor [121,145]. Liau et al. investigated the effect of DCVax-L dendritic cell vaccination on the survival of patients with glioblastoma versus patients who did not receive these vaccines and showed that patients who received this treatment had improved survival in newly diagnosed and recurrent glioblastoma [146].

#### 4.3.3. Challenges and Future Directions

Despite encouraging preclinical findings, many clinical trials have not yet resulted in significant clinical benefits for GBM patients. Issues such as tumor heterogeneity, immune evasion, and the immunosuppressive tumor microenvironment pose significant challenges [147,148]. To this end, future trials must address these complexities by incorporating validated biomarkers for patient selection and optimizing treatment regimens to maximize therapeutic outcomes while minimizing adverse effects [148]. Moreover, therapeutic resistance in GBM is further compounded by the physical and molecular complexity of the tumor. The BBB remains a major obstacle to effective drug delivery, as many therapeutic agents fail to penetrate the central nervous system in adequate concentrations [13,35]. Even when partially disrupted within the tumor core, an intact BBB in infiltrative margins continues to limit treatment efficacy [6]. In parallel, the remarkable intratumoral and intertumoral heterogeneity of GBM contributes to differential therapy responses and facilitates the emergence of resistant subclones under treatment pressure [79,107]. This adaptability underlies GBM’s notorious resistance to conventional therapies, including temozolomide and radiotherapy, further limiting long-term clinical benefits [148]. Addressing these challenges will require innovative delivery strategies, such as nanocarriers and focused ultrasound, as well as therapeutic combinations targeting multiple molecular pathways to overcome resistance mechanisms inherent to GBM biology [92,148].

#### 4.3.4. Immune Checkpoint Inhibitors (ICIs)

Although ICIs have demonstrated efficacy in other neoplasms, their impact in GBM is constrained by the tumor’s immunosuppressive microenvironment. In the CheckMate 143 trial, which compared nivolumab (anti-PD-1) with bevacizumab in patients with recurrent GBM, no significant difference in overall survival was observed [148].

#### 4.3.5. Peptide Vaccines

Peptide vaccines have been developed that target tumor-specific antigens on GBM cells. One example is Rindopepimut, which targets the epidermal growth factor receptor (EGFR) receptor gene mutation variant, EGFRvIII, which is overexpressed in 24–67% of GBM cases. Although a phase III trial revealed no significant difference in survival between the vaccine and control groups, progression-free survival was slightly higher in the vaccine group [147]. The DSP-788 vaccine targets the WT1 protein, which is overexpressed in several tumor types, including GBM, leukemias, and sarcomas. Its expression is associated with a worse prognosis. A phase III trial showed a median overall survival of 10.2 months with the vaccine and bevacizumab versus 9.4 months with bevacizumab alone, with no significant survival benefit [149].

#### 4.3.6. Dendritic Cell Vaccines

Dendritic cells, as antigen-presenting cells, activate effector T cells to attack the tumor. In a phase III trial of the DCVax-L vaccine, which uses dendritic cells loaded with autologous tumor lysate, patients who received the vaccine had a median overall survival of 19.3 months, compared with 16.5 months in the control group. This result indicates a statistically significant improvement in survival [121].

#### 4.3.7. Chimeric Antigen Receptor T Cell (CAR-T) Therapy

CAR-T therapy is a revolutionary treatment that utilizes modified T cells to identify and eliminate tumor cells. In the context of GBM, CAR-T therapy has been shown to target antigens such as IL-13Ra2, HER2, and EGFRvIII. Phase I and II clinical trials have investigated the efficacy of CAR-T therapy in GBM, with initial results that are encouraging. However, the absence of phase III trials that can definitively validate its efficacy in treating GBM underscores the need for further research and development [121,150,151].

#### 4.3.8. Stem Cells

An alternative currently in the preclinical research phase that shows promising potential is the use of stem cells. There are four distinct physiological types of stem cells: those of embryological origin (ESCs), those of adult origin (ASCs), artificially induced stem cells (iPSCs), and those found in cancerous tissues (CSCs), which function independently of external stimuli. Within ASCs, there are also mesenchymal stem cells (MSCs), neural stem cells (NSCs), and hematopoietic stem cells (HSCs). Each of these groups of cells possesses unique characteristics and properties, but they all share the ability to differentiate into other cell types, making them highly studied for the treatment of various diseases, including malignant tumors such as GBM [152].

Neoplastic tissues have the ability to secrete fibronectin, laminin, and various types of collagen, which facilitate their migration and proliferation. This protein production is regarded as a therapeutic target for stem cells. Furthermore, one of the distinguishing features of GBM is hypoxia, resulting from reduced blood flow. Transplanted stem cells benefit from this hypoxic environment, which allows them to remain in an undifferentiated state for an extended period, thereby enhancing their reparative functions [153].

One of the key challenges in the treatment of GBM is the BBB, which reduces blood flow to the brain, exacerbating the characteristic hypoxia of the tumor, and selectively restricts the passage of several substances. Stem cells, owing to their ability to traverse the BBB, can be utilized as vehicles for targeted drug delivery, thereby enhancing the direct action of therapeutic agents on the tumor [154]. Studies conducted in murine models have demonstrated the use of stem cells loaded with chemotherapeutic agents such as doxorubicin, resulting in a positive impact on animal survival [155]. Additionally, other studies have administered stem cells loaded with gold nanoparticles, which, when exposed to radiation, convert light into heat, enabling localized tumor destruction [156].

Some studies have identified a subset of glioma-specific CSCs with restorative and proliferative properties, which play a key role in tumor invasion and metastasis. Since physiological stem cells naturally migrate toward tumor cells, interactions between these two stem cell populations may reduce tumor proliferation, angiogenesis, metastasis, inflammation, and apoptosis [157,158]. Bryukhovetskiy et al. demonstrated that CSCs exhibit the strongest capacity for attracting stem cells, as they secrete various cytokines and migration inducers that influence stem cell migration following transplantation. The study found that NSCs, which are morphologically and biochemically similar to tumor cells, can be transformed into CSCs within the tumor microenvironment, promoting their proliferation. Growth factors secreted by NSCs can be exploited by the tumor to invade the brain parenchyma. On the other hand, HSCs express fewer growth factors, indicating a lower risk of neoplastic transformation, thus offering a potentially more effective treatment approach for GBM [157].

#### 4.3.9. Virology

An additional application of stem cells for antineoplastic purposes is immunotherapy through virology, which seeks to either stimulate a new immune response or strengthen the existing immune system’s defense against tumor cells [74]. Oncolytic viruses, which are non-pathogenic, selectively infect cancer cells, triggering a targeted anti-glioma cytotoxic response. This process promotes the recruitment of antigen-presenting cells (APCs), enhances the phagocytosis of dying cells, and facilitates the migration and interaction of APCs with T cells, thus bolstering the immune response against the tumor [13].

Oncolytic viruses exert an indirect immunogenic cell death mechanism by utilizing substances such as tumor-associated antigens (TAAs), PAMPs, and damage-associated molecular patterns (DAMPs) to induce apoptosis, necrosis, and autophagy in GBM cells [138]. A variety of viruses have been identified for oncolytic purposes, including parvovirus, reovirus, and HSV-1. Given the heterogeneity of GBM, it is crucial to identify the virus that best aligns with each patient’s tumor microenvironment, taking into account the immunogenic receptors they express, and to continue advancing personalized medicine [159,160]. Stem cells serve as vehicles for these viruses, ensuring that their effects are confined to GBM cells and regulating their immune clearance, dosage, and passage through the BBB [161]. Also, a study by Kiprianova et al. reported that parvovirus H1-PV is a safe and well-tolerated bioactive agent in glioblastoma patients as it exerts direct oncolytic effects on the tumor, which supports the recommendation of further clinical trials [162].

**Figure 1:**
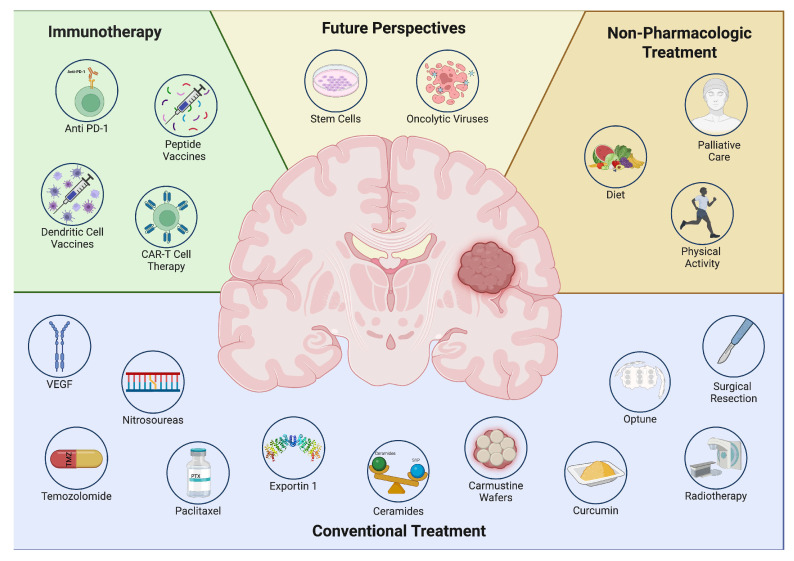
Overview of current and emerging treatments for glioblastoma. This figure illustrates different therapeutic strategies for glioblastoma. Conventional treatments include chemotherapy, radiotherapy, surgical resection, and targeted agents such as VEGF inhibitors, temozolomide, paclitaxel, nitrosoureas, carmustine wafers, curcumin, Optune, ceramides, and exportin 1. Immunotherapy approaches involve CAR-T cell therapy, dendritic cell vaccines, peptide vaccines, and anti-PD-1 therapy. Non-pharmacologic approaches such as diet, physical activity, and palliative care are also considered. Additionally, future perspectives such as stem cell therapy and oncolytic viruses represent promising avenues for glioblastoma management.

**Figure 2:**
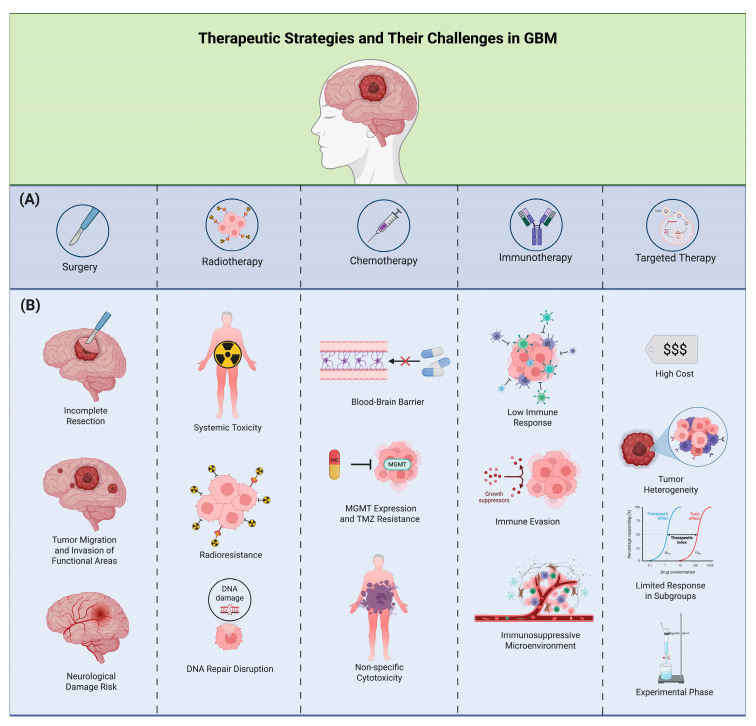
This figure illustrates the current therapeutic strategies and major challenges in the treatment of glioblastoma multiforme (GBM). Panel (**A**) shows the five main approaches used in GBM management: surgery, radiotherapy, chemotherapy, immunotherapy, and targeted therapy. Panel (**B**) outlines key limitations associated with each therapy. Surgical interventions are hindered by incomplete resection, tumor invasion into functional brain areas, and the risk of neurological damage. Radiotherapy is limited by systemic toxicity, radioresistance, and disruption of DNA repair mechanisms. Chemotherapy faces challenges such as the blood–brain barrier, resistance mediated by O6-methylguanine-DNA methyltransferase (MGMT), and the non-specific cytotoxicity of agents like temozolomide (TMZ). Immunotherapy is affected by low immune activation, immune evasion, and an immunosuppressive tumor microenvironment. Finally, targeted therapies are constrained by high costs, tumor heterogeneity, limited response in specific subgroups, and their often experimental status.

These effects make stem cells, either alone or in conjunction with oncolytic viruses, one of the most promising therapeutic alternatives, with some authors considering them to be among the only approaches that may be truly effective against GBM. However, further preclinical and clinical research is essential to substantiate the advantages of using these techniques, whether independently or in combination with conventional treatments. As can be seen, several approaches are currently being used or tested with the aim of offering hope and improving the quality of life for GBM patients (Table 1) (Figure 1 and Figure 2).

## 5. Conclusions

GBM is a highly aggressive and challenging cancer in medical oncology, with a median survival of 14.6 months and a 5-year survival rate of only 5–10%. Its high prevalence, especially in older adults, its heterogeneous behavior, and its ability to infiltrate surrounding brain tissue represent significant obstacles to current treatments. Although surgery, chemoradiotherapy, and the use of temozolomide remain the standard of care, these strategies are limited by the difficulty of achieving complete resection, tumor resistance, and physiological barriers such as the blood–brain barrier, which restricts the efficacy of many therapies. Recent advances in this field have generated hope through innovative approaches that go beyond traditional therapies. Immunotherapy, particularly through immune checkpoint inhibitors, personalized vaccines, and CAR-T cell therapies, is opening new avenues to address the immunosuppressive microenvironment of GBM. Likewise, oncolytic virotherapy not only selectively destroys tumor cells, but also stimulates antitumor immune responses, establishing itself as a promising option. Personalized medicine, exemplified by individualized multimodal immunotherapy, allows for the design of treatments adapted to the molecular and immunological profile of the tumor, optimizing therapeutic efficacy. Innovations in nanotechnology, such as NanoTherm therapy and precision radiotherapy, seek to overcome the physical and structural limitations of the tumor.

Despite these advances, GBM remains a significant medical challenge due to its complex and resilient pathophysiology, which often results in recurrence and increases the need for the implementation of palliative care in early stages. This highlights the need for ongoing investment in multidisciplinary research to integrate these new strategies into a comprehensive and personalized therapeutic framework. The future of GBM management will depend on our ability to translate these developments into tangible improvements for patients, leading to increased survival rates and improved quality of life. Continued research and clinical innovation are essential to transform the current landscape and provide hope for those facing this devastating disease.

## Figures and Tables

**Table 1:** Current treatment approaches for glioblastoma and their principal outcomes.

Treatment	Outcomes	References
**Conventional treatment**		
Surgical resection	The Stupp Protocol (maximal resection plus radiotherapy) remains the standard, with better outcomes when extensive resection is possible. Fluorescence-guided surgery with 5-ALA increases complete resection rates to about 65%.	**Batash et al., 2017; Kishwar Jafri et al., 2022.** [100,101].
TMZ	Concurrent TMZ and RT increase 2-year survival to 27.2% versus 10.9% with RT alone. TMZ with chemotherapy further improves survival.	**Alifieris et al., 2015, Schaff et al., 2023, Herrlinger et al., 2019.** [77,107,108].
PTX	Targets proliferating cells and can trigger a macrophage-mediated immune response against recurrent GBM via CD47 blockade. Albumin-bound PTX combined with TMZ shows a synergistic effect, suppressing GBM progression and improving survival in experimental models.	**Wang et al., 2022; Qu et al., 2023.** [56,110].
XPO1	XPO1 exports tumor suppressor proteins like Rb1, p27, and P53 from the nucleus. Selinexor, a selective XPO1 inhibitor, has shown efficacy in preclinical GBM models by promoting apoptosis.	**Green et al., 2015; Lassman et al., 2022.** [111,112].
Carmustine wafers	Biodegradable implants that may extend GBM survival, with one study reporting an increase in overall survival from 5.5 to 8.7 months. While a study found improved survival without significant adverse effects, further studies are needed.	**Okada et al., 2017; Zhang et al., 2014.** [113,114].
Curcumin	Curcumin has anti-inflammatory and antitumor properties. It reduces GBM cell growth, promotes apoptosis, and enhances the effects of RT and chemotherapy.	**Walker et al., 2020; Zoi et al., 2022.** [115,116].
Ceramide	Ceramides induce apoptosis, while S1P promotes tumor aggressiveness. An imbalance favoring S1P, regulated by enzymes SPHK1 and SGPP2, is linked to higher tumor grade. Inhibiting SPHK1 and increasing ceramide levels can reduce angiogenesis and improve response to TMZ.	**Karmelic, 2024; Zaibaq, 2024; Abuhusain 2013; Tea 2020.** [70,71,72,73].
RT	RT has been a GBM standard, typically delivered in 2 Gy fractions over 6 weeks (total 60 Gy). Its role in recurrent gliomas is mainly palliative, with limitations based on prior RT, tumor location, dose limits, and patient functional status.	**Hau et al., 2016; Rocha et al., 2023; Huang et al., 2020.** [120,121,122].
**Non-pharmacological treatment**		
Palliative care	Palliative care is crucial, but its timing varies due to many external and internal factors. Physical activity, though challenging due to symptoms like fatigue, may improve quality of life and mood in early stages, but evidence is limited, and its application is difficult as the disease progresses.	**Berthold, D. 2022; Rivoirard, R. 2018; Okon II, 2024; Ayotte, 2017, Capozzi 2016; Gehring K, 2018.** [123,124,125,126,127,128,129,130,131,132,133].
Diet	The ketogenic diet reduces glucose, forcing the brain to use ketones, which tumor cells cannot efficiently metabolize. Animal studies suggest this diet may enhance the effects of RT by reducing tumor angiogenesis. However, evidence on its benefits in GBM patients is contradictory, with some studies questioning its impact on prognosis or suggesting it could be ineffective.	**Li X, 2016; Montella L, 2021; Abdelwahab MG, 2012.** [131,132,133].
**Future perspectives: immunological therapy stem cells and virology**		
Optune	Interferes with mitosis by destabilizing the plasma membrane, breaking chromosomes, and causing apoptosis, as well as disrupting septin fibers and inducing autophagy. Although promising, further research is needed to understand its effects on other cellular proteins in both cancerous and normal cells.	**Davis et al., 2016.** [4].
Immune checkpoint inhibitors	ICIs show limited efficacy in GBM due to its immunosuppressive microenvironment. The CheckMate 143 trial found no overall survival benefit of nivolumab (anti-PD-1) compared to bevacizumab in recurrent GBM.	**Ng Andrew et al., 2024.** [59].
Peptide vaccines	Vaccines like Rindopepimut (targeting EGFRvIII) and DSP-788 (targeting WT1) have been developed for GBM. Although phase III trials showed slight improvements in progression-free survival or median overall survival, neither vaccine demonstrated a significant survival benefit compared to controls.	**Rong, et al, 2022; Ueda et al, 2022.** [147,149].
Dendritic cell vaccines	These vaccines activate T cells to trigger an immune response against GBM. Patients treated with these vaccines showed a median overall survival of 19.3 months versus 16.5 months in controls.	**Rocha et al., 2023.** [121].
CAR-T therapy (chimeric antigen receptor T cells)	Uses modified T cells to target GBM antigens like IL-13Ra2, HER2, and EGFRvIII. Early-phase trials show promising results, but a lack of phase III studies means more research is needed to confirm its efficacy.	**Rocha et al., 2023; Luksik et al., 2023; Montoya et al., 2024.** [121,150,151].
Stem cells	GBM’s hypoxic environment supports stem cell survival and function, while stem cells can cross the BBB, enabling targeted drug delivery directly to the tumor. Studies in animal models show stem cells loaded with chemotherapy or nanoparticles can improve survival and locally destroy tumors. However, interactions between physiological stem cells and glioma-specific CSCs are complex; NSCs may transform into CSCs and aid tumor growth, while HSCs show less risk of transformation, suggesting they could offer safer therapeutic options.	**Abadi, 2021; Herrera-Perez, 2015, Miska, 2015; Bryukhovetskiy, 2016; Pacioni, 2017**. [152,153,154,157,158].
Virology	Oncolytic viruses selectively infect and kill GBM cells while stimulating a targeted immune response. These viruses trigger cancer cell death through tumor antigens and immune signals, with stem cells acting as delivery vehicles that cross the BBB and control viral action. This combined approach shows great promise as a potentially effective GBM treatment.	**Gujar, 2019; Hamad, 2023**. [159,160].
